# Individual and Classroom Social-Cognitive Processes in Bullying: A Short-Term Longitudinal Multilevel Study

**DOI:** 10.3389/fpsyg.2019.01752

**Published:** 2019-07-31

**Authors:** Robert Thornberg, Linda Wänström, Shelley Hymel

**Affiliations:** ^1^Department of Behavioral Sciences and Learning, Linköping University, Linköping, Sweden; ^2^Department of Computer and Information Science, Linköping University, Linköping, Sweden; ^3^Educational and Counselling Psychology, and Special Education, The University of British Columbia, Vancouver, BC, Canada

**Keywords:** bullying, moral disengagement, defender self-efficacy, collective efficacy, social-cognitive theory, peer influence

## Abstract

The aim of this study was to examine whether individual and classroom collective social-cognitive processes (moral disengagement and self-efficacy) were associated with bullying perpetration among schoolchildren. An additional aim was to examine whether changes in these processes from grade 4 (Time 1) to grade 5 (Time 2) were associated with a change in bullying perpetration. Self-reported survey data were collected from 1,250 Swedish students from 98 classrooms. Results of multilevel analysis indicated that individual and classroom collective moral disengagement (CMD) were positively associated with bullying, and defender self-efficacy (DSE) was negatively associated with bullying. The effect of changes in individual moral disengagement on changes in bullying was positive, and the effects of changes in DSE and classroom collective efficacy on changes in bullying were negative. Thus, the findings demonstrate the changeability of moral disengagement, DSE and collective efficacy over time, and how these changes are linked to changes in bullying perpetration.

## Introduction

Bullying reflects a “systematic abuse of power in interpersonal relationships” (Rigby, 2008, p. 22) characterized by repeated aggression toward someone in a less powerful situation (Olweus, 2010). Bullying victimization in school is associated with a greater risk of depression, suicidal ideation and behavior, anxiety, and psychosomatic problems in childhood and adolescence (Gini and Pozzoli, 2013; Holt et al., 2015; Silberg et al., 2016), and also predicts mental health problems in adulthood (Copeland et al., 2013; Klomek et al., 2015; Lereya et al., 2015; Evans-Lacko et al., 2017; see McDougall and Vaillancourt, 2015 for a review). Although children, in general, judge bullying as morally wrong by referring to the harm it causes its victims (Thornberg, 2010; Thornberg et al., 2016), bullying still takes place among them at school (Craig et al., 2009; Chester et al., 2015), which indicates a gap between moral standards and actions. As an essentially immoral behavior with demonstrated links to delinquency and other antisocial behavior in adulthood (Bender and Lösel, 2011; Farrington and Ttofi, 2011; Olweus, 2011; Klomek et al., 2015), the presence of school bullying is a failure of moral education (Hymel et al., 2010), and a violation against the United Nations Conventions on the Rights of the Child (Lundy, 2012).

### Social-Cognitive Theory of Moral Agency

According to social-cognitive theory (Bandura, 1999, 2002, 2016), the exercise of moral agency involves the power to refrain from inhumane behavior (inhibitive morality) and the power to behave humanely (proactive morality). It includes the acquisition of moral standards and reasoning, but that is not enough. Moral agency also involves motivational and self-regulatory mechanisms in order to translate conceptions of morality into moral action. Moral self-regulation includes self-monitoring and self-evaluation linked with personal moral standards and environmental circumstances. In self-evaluation, individuals react to themselves with either self-approval for behaving in accordance with their moral standards or self-sanctions, such as feelings of guilt and remorse, for violating them. People’s beliefs in their capacity to conduct a certain moral action successfully will further influence their motivation either to act or to inhibit action, depending on how much they believe in their capacity to perform with success (also see Bandura, 1997).

A comprehensive theoretical understanding of bullying perpetration cannot be reduced to the individual characteristics of the bully, and has to include the social context (Hymel et al., 2015; Swearer et al., 2012; Salmivalli and Peets, 2018). In understanding school bullying, the most immediate context is the classroom group. Considering bullying as a group phenomenon, several scholars have emphasized the importance of examining group processes underlying bullying, including classroom dynamics, peer norms, peer socialization, group influences, collective efficacy, and peer pressure (for reviews, see Salmivalli, 2010; Hymel et al., 2015). With reference to social-cognitive theory (Bandura, 1986, 1997, 2002, 2016), how people think and act in relation to social and moral issues needs to be understood as an ongoing result of the interplay between individual and contextual factors. “Moral agency is socially situated and exercised in particularized ways depending on the life conditions under which people transact their affairs. Social cognitive theory, therefore, adopts an interactionist perspective to morality. Moral actions are the products of the interplay of personal and social influences” (Bandura, 2002, p. 115). In their 1-year longitudinal social network analysis, Caravita et al. (2014) showed that students in early adolescence, but not in late childhood, became more similar to their friends in their proneness to morally disengage. They also found that the early adolescents became more similar to their friends in bullying (Sijtsema et al., 2014). Whereas these two studies examined possible peer influence at the friendship network level, the present study was designed to examine possible social influence at the classroom level. More precisely, we focus on social-cognitive correlates at the individual and classroom level in relation to bullying perpetration.

### Individual Factors

Bandura (1999, 2002, 2016) proposed the concept *moral disengagement* as one possible factor in understanding the links between moral standards and behavior. It refers to social and psychological maneuvers that deactivate self-regulation mechanisms, thereby reducing or disengaging self-sanctions against immoral conduct (see Hymel et al., 2010 for in-depth discussion). Examples of moral disengagement mechanisms include moral justification, diffusion of responsibility, cognitively distorting the harmful consequences, dehumanization, and victim blaming. Previous research has consistently shown that bullying is associated with greater moral disengagement (see Gini et al., 2014, for a meta-analysis). However, the vast majority of studies have used a cross-sectional design (e.g., Hymel et al., 2005; Gini et al., 2011; Caravita et al., 2012; Thornberg et al., 2015). Only a few longitudinal studies have examined the association between moral disengagement and bullying over time.

In a short-term longitudinal study of Australian adolescents, conducted by Barchia and Bussey (2011a), moral disengagement predicted aggression 8 months later. Similarly, Sticca and Perren (2015) found that initial levels of moral deficiencies (i.e., an index of high moral disengagement, low moral responsibilities, and weak feelings of remorse) predicted an increase in bullying perpetration over a 2-year period among Swiss adolescents. In line with these two longitudinal studies, Wang et al. (2017) demonstrated that moral disengagement predicted bullying perpetration 6 months later among American adolescents. Although social-cognitive theory assumes an interplay or a reciprocal influence between behavior, personal factors, and external environment (the so-called triadic codetermination process; Bandura, 1986, 1997, 2016), it is still unknown whether changes in moral disengagement are related to changes in bullying perpetration.

Moral agency also depends on the belief in one’s capacity to act in accordance with moral standards (Bandura, 2016). The concept of *self-efficacy* refers to “beliefs in one’s capabilities to organize and execute the courses of action required to produce given attainments” (Bandura, 1997, p. 3). Whereas high self-efficacy motivates action if the action is in line with personal standards and goals, low self-efficacy will inhibit action (Bandura, 1997). In peer aggression and bullying situations, *defender self-efficacy*, defined as the belief in one’s capacities to intervene successfully in bullying or peer aggression to defend a victim (Thornberg et al., 2017), has been shown to be associated with greater defender behavior (Thornberg and Jungert, 2013; Doramajian and Bukowski, 2015; Peets et al., 2015; Thornberg et al., 2017), and less pro-bullying behavior (Thornberg and Jungert, 2013). Thus, children with high levels of defender self-efficacy (DSE) are more inclined to defend victims and less inclined to assist bullies or reinforce bullying by laughing or cheering on the bullies, which in turn indicates greater moral conduct in bullying situations. Of interest in the present study is whether and how DSE is related to bullying perpetration. Of additional interest is whether a *change* in DSE is associated with a *change* in bullying perpetration, as this has not yet been examined in the literature. Considering that DSE is a self-concept that makes students more inclined to defend victims and less inclined to assist bullies and reinforce bullying, it is plausible that high DSE indicates an anti-bullying stance which is often translated into action, and thus, may be associated with less bullying perpetration.

### Classroom Contextual Factors

Moral disengagement has largely been studied at the individual level (Gini et al., 2014), despite arguments for consideration at both individual and group levels (White et al., 2009). *Collective moral disengagement* refers to moral disengagement beliefs that are shared within a significant social group (Gini et al., 2015). According to Bandura (2016), collective moral disengagement (CMD) is not simply the aggregation of the moral disengagement of its individual members, but is a group-level phenomenon of perceived shared beliefs produced by the group dynamics. Therefore, as Gini et al. (2015) argue, it is important to measure its influence “through a collective measure that is independent of the personal measure, yet operates through the same set of mechanisms as the personal one” (p. 444). In the literature, this has been done by measuring and aggregating at the classroom level the students’ perceptions of the degree to which moral disengagement mechanisms are shared by their classmates (Gini et al., 2015; Kollerová et al., 2017). Classroom CMD is thus a group characteristic at the classroom level with the potential to influence group members’ attitudes and behaviors, and has been linked to aggression (Gini et al., 2015) and bullying (Kollerová et al., 2017). To date, studies investigating the association between CMD and bullying are still very few, and not one has a longitudinal design.

*Collective efficacy* is an also group-level property, one that represents a group’s capacity to work together to produce given attainments (Hymel et al., 2015). Bandura (1997) defines it as “a group’s shared belief in its conjoint capabilities to organize and execute the courses of action required to produce given levels of attainments” (p. 477). Because group functioning is more than just the sum of individual efficacies (Bandura, 1997; Fernández-Ballesteros et al., 2002; Barchia and Bussey, 2011a), its measurement should involve “aggregating members’ appraisals of their groups’ capacity as a whole” (Bandura, 1997, p. 478) rather than simply summing appraisals of one’s own individual capacities (also see Barchia and Bussey, 2011a) to cover the interactive and coordinated nature of group dynamics (Bandura, 1997; Fernández-Ballesteros et al., 2002). In peer aggression situations, *collective efficacy to stop peer aggression* refers to shared beliefs in “the ability of students and teachers to work together to stop peer aggression in schools” (Barchia and Bussey, 2011a, p. 107), and has been found to be associated with less peer aggression 8 months later among adolescents (Barchia and Bussey, 2011a).

Barchia and Bussey (2011a) argue that teachers play a significant role in inhibiting peer aggression and therefore were included in their measure of collective efficacy to stop peer aggression. Ttofi and Farrington’s (2011) meta-analysis suggests that teachers play a potentially crucial role in addressing school bullying because, among the most important bullying prevention program components associated with reductions in bullying, were: improved playground supervision, disciplinary methods, classroom management, teacher training and classroom rules. These are all aspects of the classroom context that teachers, as an “invisible hand” (Farmer et al., 2011), can orchestrate in ways that diminish or enhance the likelihood of bullying. As well, teachers have professional, and in many countries including Sweden, legal responsibilities to stop peer aggression in school (e.g., Sabia and Bass, 2017; Lunneblad et al., 2019); and because they have formal leadership roles in school classes (Hamm and Hoffman, 2016), it is reasonable to include teachers within the construct of classroom collective efficacy to stop peer aggression.

As with CMD, collective efficacy to stop peer aggression is a group-level property that reflects shared beliefs in the group’s ability to stop peer aggression, and is not adequately assessed an aggregation of individual beliefs in one’s own ability to stop peer aggression. Whereas Barchia and Bussey (2011a) examined collective efficacy at the individual level (i.e., individual perceptions of collective efficacy to stop peer aggression in school), the current study was designed to examine collective efficacy as a group characteristic, hypothesizing that a strong shared belief in the group’s ability to stop peer aggression would function as peer pressure against bullying perpetration.

### Aim and Hypotheses

The aim of the current study was to examine whether individual and classroom collective social-cognitive processes were associated with bullying perpetration among schoolchildren. A further goal was to examine whether changes in these processes from grade 4 (Time 1) to grade 5 (Time 2) were associated with concomitant changes in bullying perpetration. Gender was included as a co-variable, since previous research has found that males score higher than females on bullying (for a meta-analysis, see Cook et al., 2010). First, we hypothesized that individual and classroom CMD would be positively associated with bullying, and that changes in these processes would be positively associated with bullying changes over time. Second, we proposed a “defender efficacies as bullying refraining” hypothesis. That is, both DSE (indicating a personal anti-bullying stance often translated into anti-bullying action in bullying situations) and classroom collective efficacy to stop peer aggression (indicating anti-bullying peer pressure linked with a higher risk of social sanction and less social reward/reinforcement when bullying) are assumed to motivate individuals to refrain from bullying perpetration. Therefore, we hypothesized that DSE and classroom collective efficacy to stop peer aggression would be negatively associated with bullying, and that changes in these processes would be associated with concomitant bullying changes over time.

## Materials and Methods

### Participants

The present study is part of an ongoing longitudinal project investigating social and moral correlates of bullying in Swedish primary schools, in which students have one classroom in which most of their learning take place, and they have the same classroom teacher across most school subjects. The original sample included 2,408 fourth grade students (48% female, 52% male) from 116 primary classrooms in 74 public schools. However, 782 of those students (32%) did not participate for various reasons (599 did not obtain parental consent; 183 were absent on the day of testing or chose not to participate). Thus, at Time 1 in fourth grade, 1,626 students participated (52% female, 48% male). Out of the 1,626 students who completed the questionnaire in fourth grade, 1,344 students completed all the scales used in the current study in fifth grade as well (Time 2). A final reduction in sample size was the result of changes at the classroom level. One classroom split into two and eight classrooms merged into three classrooms from grade 4 to grade 5. In addition, twelve students changed classrooms during the study period. These classrooms and students were omitted from our study. Thus, the final sample included 1,250 students (52% females, 48% male) from 98 Swedish primary classrooms in 69 public schools who participated in the study in both fourth grade (Time 1, Age: *M* = 10.55, *SD* = 0.34) and about 1 year later in fifth grade (Time 2, Age: *M* = 11.55, *SD* = 0.32).

Although socioeconomic status was not measured at an individual level, based on a strategic sampling of schools, our sample included students from a wide range of socioeconomic backgrounds (from lower to upper-middle socioeconomic status) and socio-geographic locations (a large city, middle-sized cities, small towns, and the countryside). The majority were of Swedish ethnicity, whereas 18% had a foreign background (i.e., born in another country and/or both parents born in another country). Finally, the student composition of the 98 classrooms included in the study was highly stable; on average, 86% of students remained in the same class from fourth to fifth grade (*SD* = 10%).

### Procedure

School principals and teachers were informed of the study and gave researchers access to the classrooms. Both written, informed parental consent and student assent were obtained from all participants. Data were collected with a web-based, self-report questionnaire, which each participant completed on tablets in their regular classrooms in Grade 4 and 1 year later in Grade 5. Either a member of the research team or a teacher was present throughout the session to be available to explain the study procedure and assist participants who needed help. Teachers received instructions from the first author through a 21-min video. Team members and teachers were instructed to neither look at nor interfere with participants’ responses, but to clarify instructions, questions and words in the questionnaire if requested by participants.

### Measure

#### Individual Moral Disengagement in Peer Victimization

An 18-item scale (Bjärehed et al., 2019) was used to measure individual moral disengagement with regard to peer aggression. Students rated each item (e.g., “People who get teased don’t really get too sad about it.” “If you can’t be like everybody else, it is your own fault if you get bullied.”) on a seven-point scale (1 = “strongly disagree” to 7 = “strongly agree”). The resulting composite index of moral disengagement, with responses averaged across items, was found to be internally consistent, with Cronbach’s α of 0.82 at Time 1 and 0.89 at Time 2.

#### Collective Moral Disengagement in Peer Victimization

An 18-item scale (Bjärehed et al., 2019) was used to measure classroom CMD in peer aggression, using the same items as those measuring individual moral disengagement in order to avoid the risk of test effects due to different items when comparing individual and CMD. To capture the collective dimension of the construct (cf. Gini et al., 2015), this scale asked, “How many students in your classroom agree with the following?” and offered five response options (“none,” “about a quarter,” “about half,” “about three quarters,” “all”). An index of CMD was obtained by calculating the average score for each individual in the classroom, and then obtaining the classroom mean. Cronbach’s α was 0.91 at Time 1 and 0.93 at Time 2.

#### Defender Self-Efficacy

A six-item self-report scale was devised to measure DSE (e.g., “I feel that I’m very good at helping students who are bullied”), with responses made on a seven-point scale (1 = “strongly disagree” to 7 = “strongly agree”) and responses averaged across items. Cronbach’s α was 0.92 at both Time 1 and Time 2.

#### Collective Efficacy to Stop Peer Aggression

A Swedish translated version (Wänström et al., 2017) of Barchia and Bussey’s (2011a,b) scale was used to measure classroom collective efficacy to stop peer aggression. Students were asked “How well can the students and teachers at your school…” followed by 10 statements such as “… work together to stop bullying?”, “… work together to stop students punching each other?”, and “… work together to stop students spreading rumors about each other?” Students rated each item on a seven-point scale (from 1 = “not well” to 7 = “very well”). Cronbach’s α was 0.97 at Time 1 and 0.96 at Time 2. Collective efficacy was obtained by calculating the average score for each individual in the classroom, and then computing the classroom mean.

#### Bullying Behavior

We used an 11-item, self-report scale (Bjärehed et al., 2019) to measure bullying perpetration. Instead of providing an a priori definition of bullying, the students were asked, “Think of the past 3 months: How frequently have you done the following things toward someone who is weaker, less popular or less in charge in comparison to you?” The following behavioral items included physical (five items, e.g., “Beat or kicked someone in order to hurt him or her”), verbal (three items, e.g., “Teased and called the person mean names”), and relational bullying (three items, e.g., “Spread mean rumors or lied about the person”). For each item, students responded on a 5-point scale, from 1 = “I have never done it” to 5 = “Several times a week.” Averaging responses across items, the resulting composite index of bullying perpetration was internally consistent, with Cronbach’s α of 0.86 at Time 1 and 0.89 at Time 2.

### Statistical Models

We were interested in estimating effects of change in moral disengagement (MD) and DSE on change in bullying, as well as effects of initial levels of moral disengagement and DSE on bullying levels, while controlling for gender. This was done using a three-level regression model for which the intercept and time slope were allowed to vary across classes, as well as across individuals within classes. We estimated this model in three steps.

First, a three-level model was estimated, with the individual level variables gender, moral disengagement in fourth grade (MD_T1_), and DSE in fourth grade (DSE_T1_), as well as the time varying variable, grade (grade 4 = T1, grade 5 = T2). The intercept was allowed to vary across individuals within classes and across classes:

(Model 1) $${\text{Bully}}_{t\,i\,j}\,\text{ = }\,\beta_{0\,i\,j}+\beta_{1}\,\mathrm{Grade}+\varepsilon_{\mathrm{tij}}{\text{Bully}}_{t\,i\,j}\text{= }\,\beta_{0\,i\,j}+\beta_{1}\,\mathrm{Grade}+\varepsilon_{\mathrm{tij}}$$

$$\beta_{0\,i\,j}\,\text{ = }\,\beta_{0\,j}+\beta_{2}\,\mathrm{Gender}+\beta_{3}\,{\mathrm{MD}}_{\mathrm{T1}}+\beta_{4}\,{\mathrm{DSE}}_{\mathrm{T1}}+u_{0\,i\,j}$$

$$\beta_{0\,i\,j}\text{= }\,\beta_{0\,j}+\beta_{2}\,\mathrm{Gender}+\beta_{3}\,{\mathrm{MD}}_{\mathrm{T1}}+\beta_{4}\,{\mathrm{DSE}}_{\mathrm{T1}}+u_{0\,i\,j}\beta_{0\,j}\,\text{ = }\,\beta_{0}+v_{0\,j}\beta_{0\,j}\text{= }\,\beta_{0}+v_{0\,j}$$

where bully_tij_ is the bullying score for the i:th student in the j:th classroom at the t:th time point, β_0ij_ is the intercept for student i in classroom j, β_1_ is the time slope, ε_tij_ is a first level residual, β_0j_ is the intercept for classroom j, β_2_ to β_4_ are slopes for individual effects, u_0ij_ is a student residual, β_0_ is the mean intercept across classes, and v_oj_ is a classroom residual. All residuals are assumed to be normally distributed. In this model, we were able to assess the effects of the initial levels of moral disengagement and DSE on bullying, while controlling for gender.

In the second model, classroom level variables of CMD in grade four (CMD_T1_) and collective efficacy (CE) in grade four (CE_T1_) were added:

(Model 2) $${\text{Bully}}_{t\,i\,j}\,\text{ = }\,\beta_{0\,i\,j}+\beta_{1}\,\mathrm{Grade}+\varepsilon_{\mathrm{tij}}{\text{Bully}}_{t\,i\,j}\text{= }\,\beta_{0\,i\,j}+\beta_{1}\,\mathrm{Grade}+\varepsilon_{\mathrm{tij}}$$

$$\beta_{0\,i\,j}\,\text{ = }\,\beta_{0\,j}+\beta_{2}\,\mathrm{Gender}+\beta_{3}\,{\mathrm{MD}}_{\mathrm{T1}}+\beta_{4}\,{\mathrm{DSE}}_{\mathrm{T1}}+u_{0\,i\,j}\beta_{0\,i\,j}\text{= }\,\beta_{0\,j}+\beta_{2}\,\mathrm{Gender}+\beta_{3}\,{\mathrm{MD}}_{\mathrm{T1}}+\beta_{4}\,{\mathrm{DSE}}_{\mathrm{T1}}+u_{0\,i\,j}$$

$$\beta_{0\,j}\,\text{ = }\,\beta_{0}+\beta_{7}\,{\mathrm{CMD}}_{\mathrm{T1}}+\beta_{8}\,{\mathrm{CE}}_{\mathrm{T1}}+v_{0\,j}\beta_{0\,j}\text{= }\,\beta_{0}+\beta_{7}\,{\mathrm{CMD}}_{\mathrm{T1}}+\beta_{8}\,{\mathrm{CE}}_{\mathrm{T1}}+v_{0\,j}$$

where β_7_ and β_8_ are class level slopes. The assumptions for model 2 are the same as for model 1. In this model, we were able to assess the effects of the initial levels of CMD and collective efficacy on bullying, while controlling for gender and individual moral disengagement and self-efficacy scores.

In the third model, the time slope (grade) was allowed to vary across individuals and across classes. Individual level variables that reflected the change between grades in moral disengagement (MD_T2_-MD_T1_) and defender self-efficacy (DSE_T2_-DSE_T1_) were added on the second level. In addition, class level variables reflecting the change in collective moral disengagement (CMD_T2_-CMD_T1_) and collective self-efficacy (CE_T2_-CE_T1_) were added on the third level:

(Model 3) $${\text{Bully}}_{t\,i\,j}\,\text{ = }\,\beta_{0\,i\,j}+\beta_{1\,i\,j}\,\mathrm{Grade}+\varepsilon_{\mathrm{tij}}{\text{Bully}}_{t\,i\,j}\text{= }\,\beta_{0\,i\,j}+\beta_{1\,i\,j}\,\mathrm{Grade}+\varepsilon_{\mathrm{tij}}$$

$$\beta_{0\,i\,j}\,\text{ = }\,\beta_{0\,j}+\beta_{2}\,\mathrm{Gender}+\beta_{3}\,{\mathrm{MD}}_{\mathrm{T1}}+\beta_{4}\,{\mathrm{DSE}}_{\mathrm{T1}}+u_{0\,i\,j}\beta_{0\,i\,j}\text{= }\,\beta_{0\,j}+\beta_{2}\,\mathrm{Gender}+\beta_{3}\,{\mathrm{MD}}_{\mathrm{T1}}+\beta_{4}\,{\mathrm{DSE}}_{\mathrm{T1}}+u_{0\,i\,j}$$

$$\beta_{1\,i\,j}\,\text{ = }\,\beta_{1\,j}+\beta_{5}\,\left({\mathrm{MD}}_{\mathrm{T2}}-{\mathrm{MD}}_{\mathrm{T1}}\right)\beta_{1\,i\,j}\text{= }\,\beta_{1\,j}+\beta_{5}\,\left({\mathrm{MD}}_{\mathrm{T2}}-{\mathrm{MD}}_{\mathrm{T1}}\right)$$

$$\;+\beta_{6}\,\left(D\,S\,E_{T\,2}-D\,S\,E_{T\,1}\right)+u_{1\,i\,j}\;+\beta_{6}\,\left(D\,S\,E_{T\,2}-D\,S\,E_{T\,1}\right)+u_{1\,i\,j}$$

$$\beta_{0\,j}\,\text{ = }\,\beta_{0}+\beta_{7}\,{\mathrm{CMD}}_{\mathrm{T1}}+\beta_{8}\,{\mathrm{CE}}_{\mathrm{T1}}+v_{0\,j}\beta_{0\,j}\text{= }\,\beta_{0}+\beta_{7}\,{\mathrm{CMD}}_{\mathrm{T1}}+\beta_{8}\,{\mathrm{CE}}_{\mathrm{T1}}+v_{0\,j}$$

$$\beta_{1\,j}\,\text{ = }\,\beta_{1}+\beta_{9}\,\left({\mathrm{CMD}}_{\mathrm{T2}}-{\mathrm{CMD}}_{\mathrm{T1}}\right)\beta_{1\,j}\text{= }\,\beta_{1}+\beta_{9}\,\left({\mathrm{CMD}}_{\mathrm{T2}}-{\mathrm{CMD}}_{\mathrm{T1}}\right)$$

$$\;+\beta_{10}\,\left(C\,E_{T\,2}-C\,E_{T\,1}\right)+v_{1\,j}\;+\beta_{10}\,\left(C\,E_{T\,2}-C\,E_{T\,1}\right)+v_{1\,j}$$

where β_1ij_ is the time slope for individual i in class j, β_1j_ is the time slope for class j, and β_1_ is the mean time slope across classes, β_5_ and β_6_ are slopes for individual variables, and β_9_ and β_10_ are slopes for class level variables. Substitutions into the first row equation lead to four cross-level interactions in which Grade is a first level (varies over time) variable, the MD and DSE change variables are second level (vary over individuals) variables, and the CMD and CE change variables are third level (vary over classes) variables. This model allowed us to assess the effects of changes in individual predictors on changes in bullying, and the effects of changes in the class predictors on changes in bullying, while controlling for gender and initial levels of individual and class predictors.

Our models were evaluated by investigating Deviance (-2LL) and explained variance (R^2^). A significantly smaller Deviance and a larger proportion of explained variance indicated a better model. The chosen model was finally reduced by eliminating redundant terms (non-significant variables) from the model. The variable with the largest *p*-value was omitted first, and the model was re-estimated. If the increase in Deviance was non-significant, this model was kept, and the variable (in this new model) with the largest *p*-value was omitted, and the model was re-estimated. If the increase in Deviance was significant, the previous model was instead kept. The models were estimated using Proc Mixed in SAS. The estimation method REML (Restricted Maximum Likelihood) was used to estimate all parameters, however, the Deviance measure was calculated based on maximum likelihood (ML) estimation.

## Results

### Descriptive Statistics and Correlations

Table 1 presents descriptive statistics for individual- and classroom-level variables at grades four (T1) and five (T2). Pairwise correlations at the individual level within and between grades are presented in Table 2. As expected, bullying was positively correlated with moral disengagement and negatively correlated with DSE both within and between grades. In addition, scores on the same constructs correlated positively over time. Table 3 shows pairwise correlations at the classroom level within and between grades. The same pattern is seen here, and correlations are generally stronger at the class level. Mean bullying scores were positively correlated with CMD, and negatively correlated with collective efficacy both within and between grades, and scores were positively correlated over time.

**TABLE 1:** Means (M), standard deviations (SD), minimum and maximum observations (Min, Max) for individual- (*N* = 1250) and class level (*N* = 98) variables.

	***M***	***SD***	**Min**	**Max**
**Individual variable**				
Bullying _T1_	1.14	0.27	1.00	4.00
Moral disengagement _T1_	1.50	0.59	1.00	4.83
Defender self-efficacy _T1_	5.07	1.56	1.00	7.00
Bullying _T2_	1.16	0.32	1.00	5.00
Moral disengagement _T2_	1.41	0.62	1.00	7.00
Defender self-efficacy _T2_	4.95	1.52	1.00	7.00
**Class variable**				
Mean bullying _T1_	1.15	0.13	1.00	1.82
Collective moral disengagement _T1_	1.55	0.25	1.06	2.29
Collective efficacy _T1_	4.91	0.59	2.35	6.30
Mean bullying _T2_	1.16	0.12	1.00	1.60
Collective moral disengagement _T2_	1.53	0.24	1.03	2.29
Collective efficacy _T2_	4.78	0.62	3.00	6.09

**TABLE 2:** Correlations for individual level variables (*N* = 1250).

	**2**	**3**	**4**	**5**	**6**
(1) Bullying _T1_	0.37^∗∗∗^	−0.18^∗∗∗^	0.36^∗∗∗^	0.38^∗∗∗^	−0.15^∗∗∗^
(2) Moral disengagement _T1_		−0.21^∗∗∗^	0.22^∗∗∗^	0.46^∗∗∗^	−0.12^∗∗∗^
(3) Defender self-efficacy _T1_			−0.16^∗∗∗^	−0.18^∗∗∗^	0.44^∗∗∗^
(4) Bullying _T2_				0.50^∗∗∗^	−0.22^∗∗∗^
(5) Moral disengagement _T2_					−0.27^∗∗∗^
(6) Defender self-efficacy _T2_					1

*^∗∗∗^*p* < 0.001.*

**TABLE 3:** Correlations for class level variables (*M* = 98).

	**2**	**3**	**4**	**5**	**6**
(1) Mean bullying _T1_	0.29^∗∗^	−0.48^∗∗∗^	0.35^∗∗∗^	0.46^∗∗∗^	−0.33^∗∗^
(2) Collective moral disengagement _T1_		−0.43^∗∗∗^	0.37^∗∗∗^	0.62^∗∗∗^	−0.47^∗∗∗^
(3) Collective efficacy _T1_			−0.22^*^	−0.46^∗∗∗^	0.50^∗∗∗^
(4) Mean bullying _T2_				0.56^∗∗∗^	−0.49^∗∗∗^
(5) Collective moral disengagement _T2_					−0.70^∗∗∗^
(6) Collective efficacy _T2_					1

^*^p < 0.05, ^∗∗^*p* < 0.01, ^∗∗∗^*p* < 0.001.

### Multilevel Analyses

Table 4 displays estimates and standard errors from the multilevel analyses for models 1, 2 and 3, and for the final model. All variables, except grade and gender, were grand mean centered. The intraclass correlation (ICC) for the empty model was 0.07, indicating that 7% of the total variance in bullying was between classes. As shown in model 1, initial levels of moral disengagement were positively associated with bullying, and initial levels of DSE were negatively associated with bullying, when controlling for gender. In addition, boys scored higher than girls. The class intercept variance was significant, indicating that the classes varied in their mean bullying scores. The individual variables explained 9.4% of the variance in bullying.

**TABLE 4:** Estimates (Est) and standard errors (SE) from multilevel regression analyses of models (1), (2), and (3) with bullying as the dependent variable.

**Predictor**	**Model 1 Est (SE)**	**Model 2 Est (SE)**	**Model 3 Est (SE)**	**Final model Est (SE)**
**Time level**				
Grade	0.02 (0.01)	0.02 (0.01)	0.02^*^(0.01)	0.02^*^(0.01)
**Individual level**				
Gender	0.03^*^(0.01)	0.03^*^(0.01)	0.02 (0.01)	
MD_T1_	0.13^∗∗∗^(0.01)	0.12^∗∗∗^(0.01)	0.18^∗∗∗^ (0.01)	0.19^∗∗∗^ (0.01)
DSE_T1_	−0.02^∗∗∗^ (0.00)	−0.02^∗∗∗^(0.00)	−0.02^∗∗∗^(0.00)	−0.02^∗∗∗^(0.00)
**Classroom level**				
CMD_T1_		0.08 (0.04)	0.07 (0.04)	0.10^∗∗^(0.03)
CE_T1_		−0.02 (.02)	−0.02 (0.02)	
**Cross-level interactions**				
Grade × MD_T2_-MD_T1_			0.22^∗∗∗^(0.01)	0.22^∗∗∗^(0.01)
Grade × DSE_T2_-DSE_T1_			−0.01^*^(0.01)	−0.01^*^(0.01)
Grade × CMD_T2_-CMD_T1_			0.02 (0.05)	
Grade × CE_T2_-CE_T1_			−0.05^∗∗^(0.02)	−0.05^∗∗^(0.02)
**Variance**				
Class intercept	0.00^∗∗∗^(0.00)^a^	0.00^∗∗^(0.00)^b^	0.00^∗∗^(0.00)^*c*^	0.00^∗∗^(0.00)^c^
Class slope			0.00 (0.00)	
Individual intercept	0.00 (0.00)	0.00 (0.00)	0.00 (0.00)	0.00 (0.00)
Individual slope			0^d^	
Within individual	0.08^∗∗∗^(0.00)	0.08^∗∗∗^(0.00)	0.07^∗∗∗^(0.00)	0.07^∗∗∗^(0.00)
Deviance	719.7	710.5	384.4	389.4
ICC	0.07			
R^2^	0.09	0.10	0.22	0.22

All predictors (except for grade and gender) were grand mean centered. Grade (0 = fourth grade, 1 = fifth grade). Gender (0 = girl, 1 = boy). ^*^p < 0.05, ^∗∗^*p* < 0.01, ^∗∗∗^*p* < 0.001; ^*a*^0.004 (0.001), ^*b*^0.004 (0.001), ^*c*^0.002^∗∗^(0.0008). ^*d*^The grade slope variance between individuals reduced to zero in model 3, and was omitted. MD, moral disengagement, DSE, defender self-efficacy, CMD, collective moral disengagement, CE, collective efficacy.

As class level variables were added in model 2, initial levels of moral disengagement and DSE were still significantly associated with bullying. None of the initial class level variables was significantly related to bullying. The variables explained 10% of the variance in bullying. The Deviance measure decreased from 719.7 to 710.5, which is a significant decrease [x^2^(2) = 9.2, *p* < 0.05], indicating that model 2 is preferred over model 1.

When change variables were added in model 3, the initial levels of moral disengagement and DSE were still associated with bullying. In addition, three of the interactions, as well as Grade, were significantly associated with bullying. The variables explained 21.8% of the variance in bullying, and the decrease in Deviance was significant [x^2^(5) = 326.1, *p* < 0.001], indicating that model 3 is preferred over model 2.

In model 3, the variable with the largest *p*-value was the interaction, Grade × CMD_T2_-CMD_T1_ (*p* = 0.773). Model 3 was therefore re-estimated with this variable omitted, and the increase in Deviance was not significant [x^2^(1) = 0.1, *p* > 0.05]. The variable with the largest *p*-value in the new model was CE_T1_ (*p* = 0.153) and the model was re-estimated omitting this variable. The increase in Deviance was not significant [x^2^(1) = 2.1, *p* > 0.05]. Gender had the largest *p*-value in the new model (*p* = 0.092) and was omitted. The Deviance increase was not significant [x^2^(1) = 2.8, *p* > 0.05]. The variable with the largest *p*-value in this model (Grade × DSE_T2_-DSE_T1_: *p* = 0.029) could not be omitted without resulting in a worse fitting model [x^2^(1) = 4.8, *p* < 0.05]. The final model is thus the model in which the interaction term Grade × CMD_T2_-CMD_T1_, CE_T1_, and Gender were omitted. Model results are shown in the last column in Table 4.

As shown, Grade was significant, indicating that bullying scores increased over time. Consistent with prior research, initial levels of individual moral disengagement and initial levels of CMD scores were positively associated with bullying, and initial levels of individual DSE scores were negatively associated with bullying. In addition, the positive effect of change in moral disengagement on change in bullying (Grade × MD_T2_-MD_T1_) was significant, as were the negative effects of change in DSE on change in bullying (Grade × DSE_T2_-DSE_T1_), and change in collective efficacy on change in bullying (Grade × CE_T2_-CE_T1_). The variables explained 21.8% of the variance in bullying. The increase in Deviance from model 3 to the final model was non-significant [x^2^(3) = 5.0, *p* > 0.05], indicating that the final model is the preferable model.

In order to understand and interpret the interaction effects, we computed simple slopes. The simple slope for children with high values (one standard deviation above the mean) on MD_T2_-MD_T1_ (an increase of 0.54; −0.09+0.63 = 0.54) was 0.16 (*p* < 0.001), and the simple slope for children with low values (one standard deviation below the mean) on MD_T2_-MD_T1_ (a decrease of 0.72; −0.09−0.63 = −0.72) was −0.12 (*p* < 0.001). In these calculations, the value −0.09 is the mean value of MD_T2_-MD_T1_ and the value 0.63 is the standard deviation. The simple slope for high values on DSE_T2_-DSE_T1_ (an increase of 1.52; SD_DSET2–DSET1_ = 1.63) was −0.01 (*p* > 0.05), and the simple slope for low values (a decrease of 1.76) was 0.04 (*p* < 0.01). Finally, the simple slope for high values on CMD_T2_-CMD_T1_ (an increase of 0.43; SD_CET2–CET1_ = 0.56) was −0.00 (*p* > 0.05), and the simple slope for low values (a decrease of 0.69) was 0.05 (*p* < 0.001).

**FIGURE 1:**
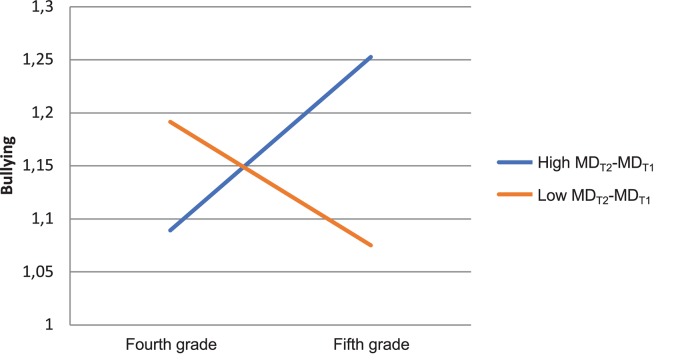
Interaction effect on bullying change: Individual moral disengagement (MD) change × grade.

**FIGURE 2:**
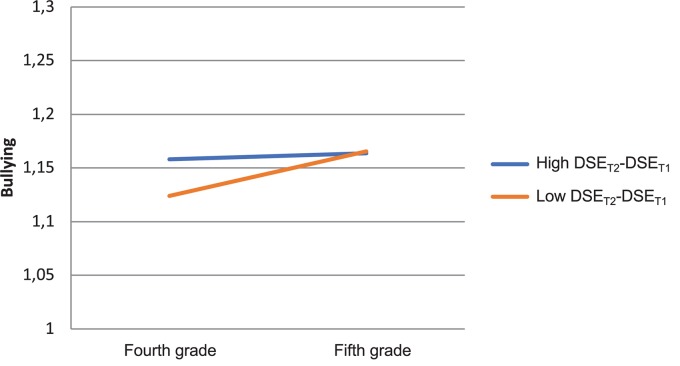
Interaction effect on bullying change: Defender self-efficacy (DSE) change × grade.

**FIGURE 3:**
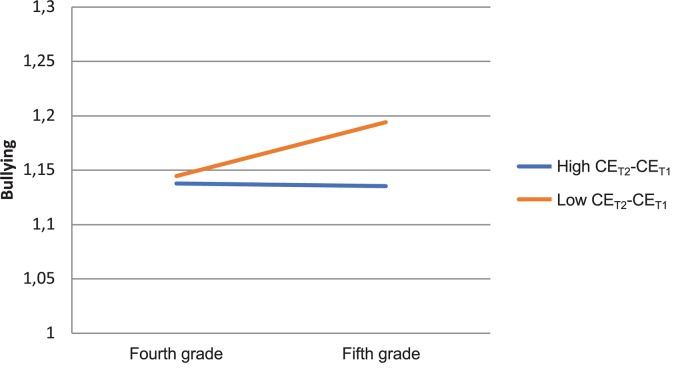
Interaction effect on bullying change: Collective efficacy (ce) change × grade.

To illustrate these slopes, we plotted them in Figures 1–3 for “typical” children. The change variables (MD_T2_-MD_T1_, DSE_T2_-DSE_T1_, and CMD_T2_-CMD_T1_) were negatively correlated with the initial value variables (MD1, DSE1, and CMD1), as might be expected. A median split for each change variable resulted in datasets consisting of children with above the median values on each change variable, and below the median values on each change variable, respectively. We used the means of the initial variables in these datasets as inputs in the equation for the final model, to plot the interaction graphs. Thus, in Figure 1, we can see that children who increased in MD (blue line) also increased in bullying. These children had typically below average initial values of MD and above average values on DSE (MD_T1_ = 1.24, DS_T1_ = 5.22, CMD_T1_ = 1.49). Children who decreased in moral disengagement (red line) also decreased in bullying and they typically had above average initial values on MD, and below average values on DSE (MD_T1_ = 1.75, DSE_T1_ = 4.93, CMD_T1_ = 1.56).

In Figure 2, we can see that children who increased in DSE (blue line) did not significantly increase in bullying. These children typically had above average initial values on MD and below average values on DSE (MD_T1_ = 1.79, DSE_T1_ = 4.46, CMD_T1_ = 1.55). Children who decreased in DSE (red line) increased in bullying, and they typically had below average initial values on MD and above average on DSE (MD_T1_ = 1.47, DSE_T1_ = 5.69, CMD_T1_ = 1.56). Finally, in Figure 3, we can see that children in classrooms that increased in CE (blue line) did not significantly increase or decrease in bullying, and they typically had below average initial values on MD and DSE (MD_T1_ = 1.48, DSE_T1_ = 5.03, CMD_T1_ = 1.55). Children in classrooms that decreased in collective efficacy (red line) increased in bullying, and they typically had above average initial values on MD and DSE (MD_T1_ = 1.52, DSE_T1_ = 5.11, CMD_T1_ = 1.55).

## Discussion

The present study is, as far as we know, the first study to examine whether changes in individual moral disengagement, DSE, classroom CMD, and classroom collective efficacy to stop peer aggression were associated with bullying changes over time in a multilevel model. Our findings contribute to the social-cognitive literature on bullying (e.g., Thornberg and Jungert, 2013; Gini et al., 2014, 2015) by showing that changes in bullying perpetration among schoolchildren were positively associated with changes in individual moral disengagement and negatively associated with changes in DSE and classroom collective efficacy to stop peer aggression over a 1-year period. Whereas previous longitudinal studies suggest that individual moral disengagement predicts greater aggression (Barchia and Bussey, 2011a) and bullying (Wang et al., 2017; cf. Sticca and Perren, 2015) over time, our findings contribute to the literature by showing that children who decreased in individual moral disengagement (more than the average individual) became less inclined to bully others, and children who increased in individual moral disengagement became more inclined to bully others.

Our study is also the first to examine possible associations between DSE and bullying, and our findings demonstrate that greater DSE in fourth grade was linked to less bullying from grade four to five. This is an important addition to the literature in demonstrating that DSE not only increases the likelihood of defending, as shown in previous studies (Thornberg and Jungert, 2013; Doramajian and Bukowski, 2015; Peets et al., 2015; Thornberg et al., 2017), but also lowers the risk of engaging in bullying perpetration. This finding also supports our initial hypothesis that DSE reflects a personal anti-bullying stance often translated into anti-bullying action in bullying situations – including moral cognition and self-regulatory skills that increase the power not only to defend (proactive morality) but also to refrain from bullying perpetration (inhibitive morality; cf. Bandura, 2016). Future research should examine the associations between DSE, self-regulation and anti-bullying attitudes. However, whereas children who decreased in DSE became more inclined to bully others, those who increased in DSE did not change their bullying perpetration. Thus, the findings suggest that when a particular level of bullying perpetration has been established, an increase in DSE does not seem to have influence. The bivariate correlations revealed that bullying is more strongly associated with moral disengagement than DSE, and change in moral cognition might be more important than change in DSE to explain change in bullying perpetration. At the same time, students who decrease in DSE tend to be more inclined to bully others, which once again supports our “defender efficacies as bullying refraining” hypothesis.

Although moral disengagement and DSE tend to be developed into trait-like habitual patterns (Bandura, 1997, 2016), they should not be considered as fixed, stable and static personality traits (cf. Kuilman et al., 2019) like, for instance, callous-unemotional traits (Frick et al., 2018). In line with previous studies of moral disengagement (e.g., Caravita et al., 2014), our findings reveal the changeability of moral disengagement and DSE, which in turn suggests the ability to learn these individual characteristics. In other words, moral disengagement and DSE seem to be individual characteristics that could be influenced and changed in late childhood. The current findings demonstrate the interplay between personal influences (moral disengagement and DSE) and behavioral influences (bullying) over time, and thus support social-cognitive theory (Bandura, 1997, 2016), as it assumes the changing nature of these processes through triadic codetermination.

Finally, whereas Barchia and Bussey (2011a) found that individual perceptions of collective efficacy to stop peer aggression among adolescents were linked with lower aggression 8 months late, our findings show that children who belong to a classroom that decreases in collective efficacy to stop peer aggression become more inclined to bully others. These findings further support the triadic codetermination of social-cognitive theory (Bandura, 1997, 2016) by indicating an interplay of environmental influences and behavioral influences, as well as our proposed “defender efficacies as bullying refraining” hypothesis. Not only DSE, as discussed above, but also classroom collective efficacy to stop peer aggression seem to inhibit schoolchildren from bullying others. A possible explanation is that higher levels of collective efficacy to stop peer aggression could be considered as a group process that produces anti-bullying peer pressure associated with an increased risk of social sanction and less social reward/reinforcement toward group members who perpetrate bullying. Children’s individual beliefs in the collective ability of students and teachers to work together to stop peer aggression have been linked to defending in peer aggression (Barchia and Bussey, 2011b). It is plausible to assume that higher levels of collective efficacy to stop peer aggression are associated with less reinforcing and greater defending at the classroom level, which in turn have been linked to less bullying (Kärnä et al., 2010; Salmivalli et al., 2011; Nocentini et al., 2013; Thornberg and Wänström, 2018). Future studies are required to understand better the possible associations between collective efficacy to stop peer aggression, anti-bullying norms, perceived peer-pressure, and the prevalence of bullying perpetration and various bystander reactions at classroom level.

Whereas previous cross-sectional studies have shown that classroom CMD is associated with greater aggression (Gini et al., 2015) and bullying (Kollerová et al., 2017), a change in classroom CMD was not found to be associated with a change in bullying over time in the current study. A possible explanation is that the early levels of CMD might have a more long-term effect on bullying, and that change in an individual’s moral disengagement is more influential in increasing or decreasing inhumane behaviors such as bullying. The cross-sectional correlations between classroom CMD and bullying prevalence at both Time 1 and Time 2, and the longitudinal correlations between classroom CMD and bullying prevalence were all significant at the classroom level. Moreover, the intercept of the bullying trend was positively associated with the initial levels of individual and classroom CMD in the final multilevel model. In other words, greater CMD in fourth grade was linked to more bullying across grades four and five (i.e., the general bullying level), suggesting that CMD functions as a protective classroom group process that lowers the risk of bullying perpetration.

### Limitations

Despite the many strengths of this study, such as the longitudinal multilevel design, some limitations should be noted. First, changes were measured during a limited period of 1 year at only two time points. Although we demonstrated that a change in bullying was associated with a change in moral disengagement, DSE and classroom collective efficacy to stop peer aggression during that 1-year period, future studies should expand these analyses by adopting a longer longitudinal design with several time points to make better predictions about the development of bullying and gain a more complete insight into the dynamics over time.

Second, we used self-report data, which are vulnerable to under- and over-estimation. To decrease the risk of under-reporting bullying perpetration, we used a bullying scale that did not include the term “bullying.” Still, self-reported data are vulnerable to various biases, such as social desirability, memory distortion, careless marking, and intentionally exaggerated responses.

Third, the findings could be critically discussed in terms of norm uncertainty/ambiguity and pluralistic ignorance (cf. Veenstra et al., 2018). In the social psychological literature, pluralistic ignorance often refers to “the beliefs that one’s attitudes and judgments are different from those of others, even though one’s public behavior is identical” (Prentice and Miller, 2003, p. 585). Individuals might privately reject a norm at the same time as they publicly conform to this norm based on an incorrect assumption that it is shared in the peer group. One way of testing this would be to aggregate individual moral disengagement and DSE at classroom level, conceptualized as *prescriptive (or injunctive) norms* of the school class (cf. Veenstra et al., 2018). In the literature, aggregating individual moral disengagement is termed *class moral disengagement* (Pozzoli et al., 2012; Thornberg et al., 2017). A drawback with this procedure of measuring prescriptive norms to “represent perceived moral rules of the peer group” (Veenstra et al., 2018, p. 49), however, is that individual classmates’ moral disengagement tendencies (like attitudes) might be more or less invisible to other members in the school class, and thus a less powerful group influence as compared to CMD.

Just as individual attitudes have been aggregated to assess prescriptive norms, individual moral disengagement and DSE can also be aggregated at the classroom level. For reasons outlined in the introduction, we considered the assessment of group functioning to be greater than the sum of individual perceptions or beliefs, and argued that the collective indices employed in the present study more accurately assess group-level shared beliefs, as proposed by Bandura (1997) and therefore assessed CMD and collective self-efficacy. Nevertheless, we reran the multilevel analyses described above, replacing our group-level indices with classroom aggregations of the individual variables of moral disengagement and DSE. Results indicated that the individual variables were still significant as well as the effects of the changes in individual moral disengagement and DSE on the change in bullying. Unlike the findings presented for our final model, however, the effect of the initial class mean of moral disengagement on bullying across the grades, and the effect of the change in the class mean of DSE on the change in bullying were not significant. One possible explanation for the different findings may be that the later model consists of the very same data at the individual level and at the classroom (aggregated) level (same scales), whereas the final model supported in our study was based on distinct assessments of individual and collective assessments (different scales). An alternative or complementary possibility is that classroom CMD (initial level) and collective efficacy to stop peer aggression (change over time) constitute more powerful group influences on bullying than classroom aggregations of individual moral disengagement and DSE.

Finally, a note of caution needs to be sounded considering generalization of the findings. The present sample consisted of students in Swedish schools and considered a very limited age span. Future research should examine the variables and their associations found in the present study in other samples of students of different age levels and in various national and cultural contexts. In addition, further research should examine whether the associations in the current findings are consistent or vary across different friendship networks nested in school classes and in relation to both perceived and sociometric popularity.

### Practical Implications

The current findings have practical implications for anti-bullying programs in schools. In accordance with previous research on moral disengagement and bullying (Gini et al., 2014), anti-bullying programs should develop components that expose and inhibit moral disengagement and facilitate the development of moral agency. We agree with Pozzoli et al. (2012), in suggesting that future research consider “a randomized control trial aimed at encouraging educators to make efforts to address these distortions in morality in order to favor children’s moral engagement and promote the understanding of responsibility” (p. 386). Using children’s literature to discuss moral disengagement, increase awareness about bullying, teach appropriate social skills, and encourage defending in bullying situations has been found to be promising in decreasing both moral disengagement and victimization among elementary school students (Wang and Goldberg, 2017).

Teachers play a significant socialization role for moral disengagement in how they respond to bullying in school. Campaert et al. (2017) found that students who reported that their teachers responded to bullying with high-level disciplinary sanctions and victim support were less inclined to morally disengage and less likely to bully. In contrast, teacher non-intervention was associated with greater moral disengagement and bullying. The intertwined changeability in our findings highlights the importance of addressing and decreasing both the cognitive influence of moral disengagement and the behavioral influence of bullying, as these seem to interplay. Even though change in classroom CMD was not linked to change in bullying, its initial level was associated with higher levels of bullying. This suggests that teachers may benefit from professional training in influencing development at the group (classroom or school) level in order to prevent CMD in the first place and to promote a moral climate of engagement and social responsibility from the very first days as a part of their classroom management.

Another important part of the development of a protective moral climate is, as suggested by our findings, to promote and maintain a high level of collective efficacy to stop peer aggression in school classes. Teachers’ efforts in building warm and supportive relations with students (Bouchard and Smith, 2017), creating an authoritative classroom climate (Thornberg et al., 2018), and effectively preventing and intervening in bullying (Ttofi and Farrington, 2011) are crucial. Bullying victimization has been found to be lower in classes where a high proportion of students state that they are aware of the school rules and that adults intervene against bullying (Låftman et al., 2017). Espelage et al. (2014) found that schools in which staff and teachers reported a greater commitment to prevent bullying had lower prevalence of bullying; Olsson et al. (2017) reported that schools which scored high in good order, cohesion, and mutual trust tended to have fewer problems with bullying. A part of classroom collective efficacy to stop peer aggression is that students trust teachers and teachers collaborate with students to prevent bullying and other forms of peer aggression. In other words, teachers need to be committed and active in counteracting bullying at the same time as they involve and engage students in their efforts to support an anti-bullying culture. A strong collective efficacy to stop peer aggression might in turn encourage students to enhance their own DSE, as there is an interplay between self-efficacy and collective efficacy (Bandura, 1997). Considering that initial levels of CMD were linked to bullying across the grades and that decreases in collective efficacy to stop peer aggression were linked to increases in bullying, school and classroom efforts to prevent CMD and develop high collective efficacy to stop peer aggression should be designed and delivered as early as possible.

Findings from a recent meta-analysis (Lee et al., 2015) indicate that consideration of group dynamics can enhance the efficacy and impact of school-based anti-bullying interventions, and a recent review by Hymel et al. (2015) points to a number of ways in which positive group processes can be fostered in the classroom context, with teachers playing a critical role in such efforts. Support for the efficacy of such a focus come from Farmer et al. (2011, 2013), who have developed the SEALS program to enhance teachers’ understanding of group processes in creating positive and supportive classroom contexts. As well, Choi et al. (2011a, b) have shown that greater experience with cooperative learning in classrooms is associated with increased prosocial behavior and decreased aggressive behavior among students.

## Conclusion

Overall, the present findings contribute novel insights to the literature on moral disengagement, self-efficacy and collective efficacy and their links to school bullying. Due to the multilevel approach, we were able to examine children’s and school classes’ bullying growth curves and their correlates. Specifically, the current study reveals the changeability of moral disengagement, DSE and classroom collective efficacy to stop peer aggression over time, and how this changeability is linked to changes in bullying perpetration. We also found that, although CMD was not associated with a change in bullying, it was associated with the general level of bullying. Consistent with a social psychological stance (e.g., Salmivalli, 2010; Hymel et al., 2015) and a social-ecological perspective on school bullying (e.g., Espelage and Swearer, 2004; Swearer et al., 2012; Trach et al., 2018), results of the present study contribute to a growing body of research underscoring the importance of addressing contextual as well as individual factors in efforts to reduce school bullying, with group processes manifested as CMD and collective efficacy to stop peer aggression being shown as critical aspects of the classroom context.

## Data Availability

The datasets generated for this study are available on request to the corresponding author.

## Ethics Statement

This study was approved by the Regional Ethical Review Board in Linköping. Written informed consent was obtained from the parents of all participants.

## Author Contributions

All authors listed have made a substantial, direct and intellectual contribution to the work, and approved it for publication.

## Conflict of Interest Statement

The authors declare that the research was conducted in the absence of any commercial or financial relationships that could be construed as a potential conflict of interest.
